# Supplementary material for: DMRforPairs: identifying Differentially Methylated Regions between unique samples using array based methylation profiles
Source: BMC Bioinformatics. 2014 May 15;15:141. doi: 10.1186/1471-2105-15-141 (PMC4046028; doi:10.1186/1471-2105-15-141)
Supplement: Additional file 2 — DMRforPairs output for the comparison of A431-MCF7 and NA17018-NA17105. Please start from the HTML files in each folder. Available via the BMC Bioinformatics website. [file 1471-2105-15-141-S2.zip › 1394847754114233_MOESM2_ESM/A431_MCF7/figures/14920.pdf]

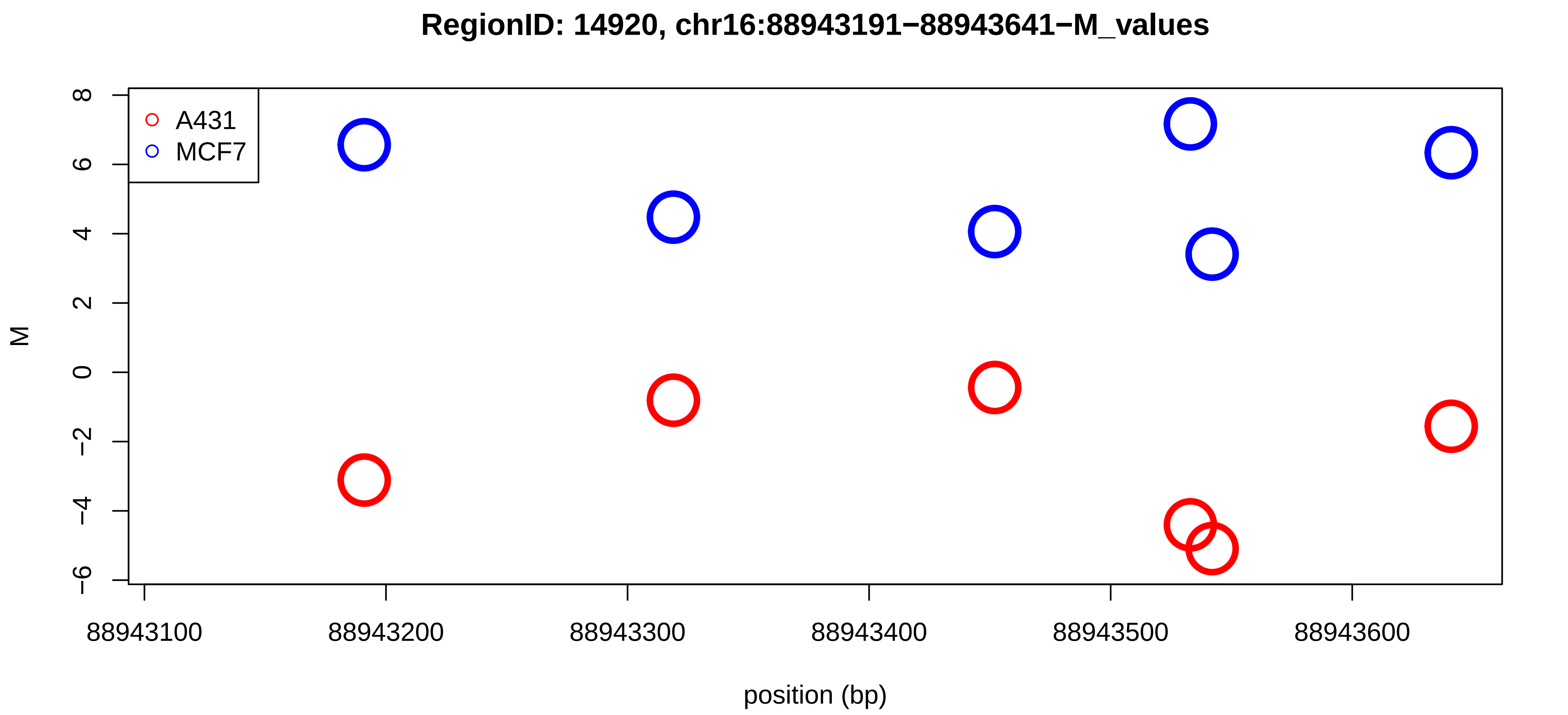

RegionID: 14920, chr16:88943191−88943641−M_values
A431
MCF7
M
8
6
4
2
0
−2
−4
−6
88943100
88943200
88943300
88943400
88943500
88943600
position (bp)

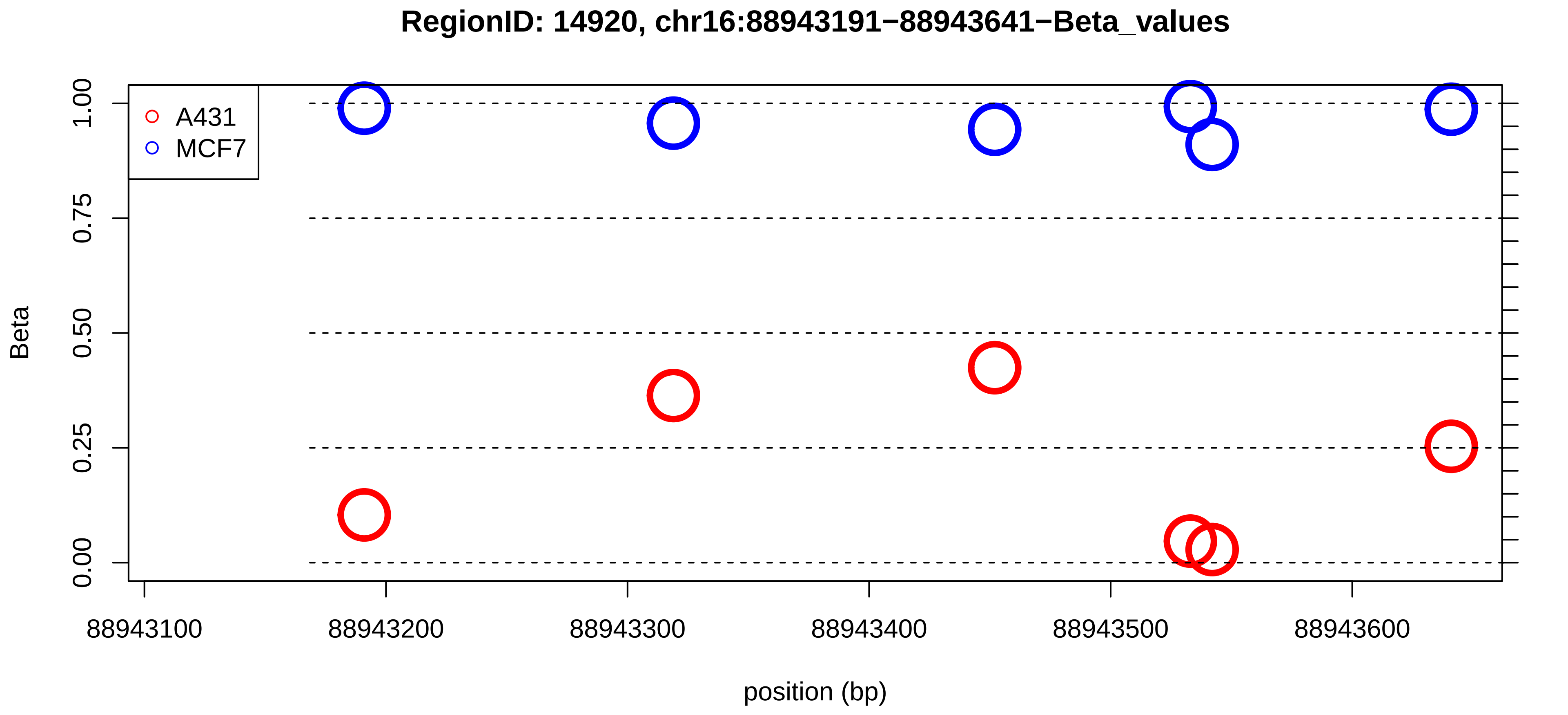

RegionID: 14920, chr16:88943191−88943641−Beta_values
A431
MCF7
Beta
1.00
0.75
0.50
0.25
0.00
88943100
88943200
88943300
88943400
88943500
88943600
position (bp)

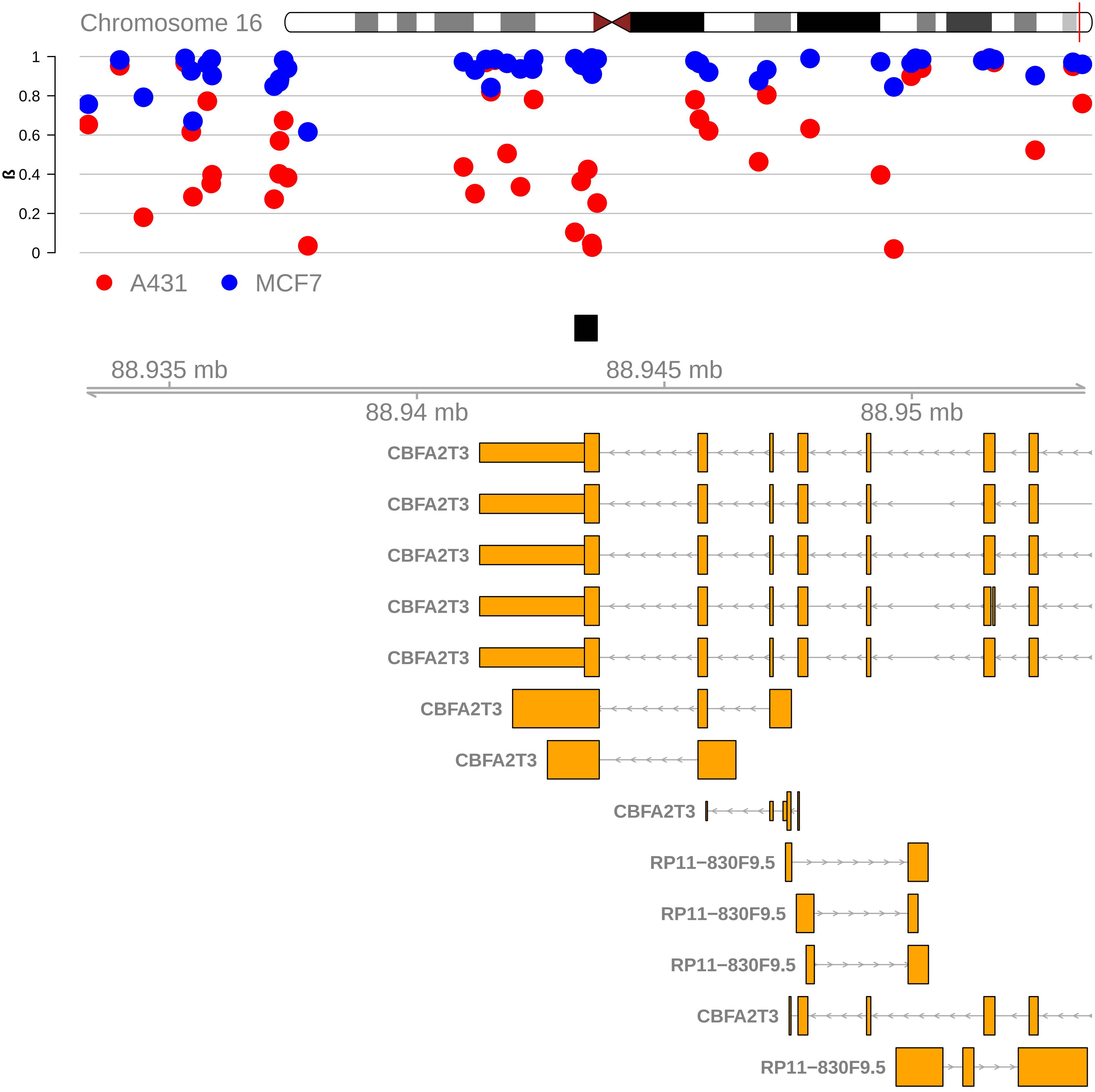
Chromosome 16
1
0.8
0.6
ß 0.4
0.2
0
A431
MCF7
88.935 mb
88.945 mb
88.94 mb
88.95 mb
CBFA2T3
CBFA2T3
CBFA2T3
CBFA2T3
CBFA2T3
CBFA2T3
CBFA2T3
CBFA2T3
RP11−830F9.5
RP11−830F9.5
RP11−830F9.5
CBFA2T3
RP11−830F9.5
